# RETRACTION: Evaluation of Bevacizumab Biosimilar on Wound Healing Complications in Patients with Colorectal Cancer Undergoing Endoscopic Mucosal Resection: A Systematic Review and Meta‐Analysis in Anorectal Medicine

**DOI:** 10.1111/iwj.70522

**Published:** 2025-04-21

## Abstract

**RETRACTION:** LiY. , MeiZ. , ShiL. , WanY. , ZhouX. , ZengT. , LiuY. , YangJ. Y. , and ShiZ. , “Evaluation of Bevacizumab Biosimilar on Wound Healing Complications in Patients with Colorectal Cancer Undergoing Endoscopic Mucosal Resection: A Systematic Review and Meta‐Analysis in Anorectal Medicine,” International Wound Journal 21, no. 1 (2024): e14638, 10.1111/iwj.14638.38272807 PMC10805537

The above article, published online on 23 January 2024, in Wiley Online Library (http://onlinelibrary.wiley.com/), has been retracted by agreement between the journal Editor in Chief, Professor Keith Harding; and John Wiley & Sons Ltd. Following an investigation by the publisher, all parties have concluded that this article was accepted solely on the basis of a compromised peer review process. The editors have therefore decided to retract the article. The authors did not respond to our notice regarding the retraction.

**RETRACTION:** Y. Li , Z. Mei , L. Shi , Y. Wan , X. Zhou , T. Zeng , Y. Liu , J. Y. Yang , and Z. Shi , “Evaluation of Bevacizumab Biosimilar on Wound Healing Complications in Patients with Colorectal Cancer Undergoing Endoscopic Mucosal Resection: A Systematic Review and Meta‐Analysis in Anorectal Medicine,” International Wound Journal 21, no. 1 (2024): e14638, 10.1111/iwj.14638.38272807 PMC10805537

The above article, published online on 23 January 2024, in Wiley Online Library (http://onlinelibrary.wiley.com/), has been retracted by agreement between the journal Editor in Chief, Professor Keith Harding; and John Wiley & Sons Ltd. Following an investigation by the publisher, all parties have concluded that this article was accepted solely on the basis of a compromised peer review process. The editors have therefore decided to retract the article. The authors did not respond to our notice regarding the retraction.

